# The relationship between triglyceride-glucose index, triglyceride-glucose-body mass index, and the severity of hepatic steatosis and liver fibrosis in patients with MASLD: a cross-sectional study

**DOI:** 10.3389/fnut.2026.1740308

**Published:** 2026-02-26

**Authors:** Xingye Wu, Jiacheng Mo, Jinming Yu, Shisi Zhang, Dan Zheng, Xiaoying Chen, Ruibing Qi, Jiaqin Jiang, Kun Ye, Zhengming Li

**Affiliations:** 1 Department of Endocrinology and Metabolism, Guangxi Academy of Medical Sciences and the People's Hospital of Guangxi Zhuang Autonomous Region, Nanning, China; 2 Information Network Management Center, The Health Governance and Smart Medical Engineering Research Center of Guangxi Zhuang Autonomous Region, Guangxi Academy of Medical Sciences and the People's Hospital of Guangxi Zhuang Autonomous Region, Nanning, China; 3 Guangxi Clinical Research Center for Chronic Kidney Disease, Guangxi Academy of Medical Sciences and the People's Hospital of Guangxi Zhuang Autonomous Region, Nanning, China

**Keywords:** insulin resistance, liver fibrosis, metabolic dysfunction-associated steatotic liver disease, triglyceride-glucose index, triglyceride-glucose-body mass index

## Abstract

**Objective:**

This study aims to investigate the relationship between the triglyceride-glucose (TyG) index and the triglyceride-glucose-body mass (TyG-BMI) index and the severity of hepatic steatosis and liver fibrosis in patients with metabolic dysfunction-associated steatotic liver disease (MASLD), providing more accurate metabolic indicators for non-invasive screening.

**Methods:**

A retrospective analysis was conducted on 7,035 subjects who completed Fibrotouch testing. Multivariate logistic regression analysis was used to assess the correlation between the TyG index and TyG-BMI index and the severity of hepatic steatosis (mild, moderate, severe) and hepatic fibrosis. Quartile grouping analysis and ROC curve assessment were also used to evaluate predictive performance.

**Results:**

After adjusting for potential confounding factors, both the TyG index and the TyG-BMI index were significantly positively correlated with the severity of MASLD (both *p* < 0.001). The association was stronger for TyG-BMI, with the highest quartile (Q4) having a 239.41-fold higher risk of hepatic steatosis compared to the lowest quartile (Q1; OR = 239.41, 95% CI: 170.99–335.21). For liver fibrosis, only TyG-BMI maintained independent association (OR = 1.029, 95% CI: 1.025–1.033). ROC analysis showed that TyG-BMI had significantly better predictive performance for MASLD than TyG (AUC: 0.908 vs. 0.774).

**Conclusions:**

The TyG-BMI index is a strong predictor of MASLD severity and liver fibrosis, with predictive performance superior to that of the traditional TyG index. It can serve as an effective tool for clinical screening of high-risk populations for MASLD.

## Introduction

Due to changes in diet and lifestyle, an aging population, and the increasing prevalence of diabetes and obesity, non-alcoholic fatty liver disease (NAFLD) has become the most common chronic liver disease worldwide (1, 2). Among these, metabolic dysfunction-associated steatotic liver disease (MASLD) has emerged as a new disease subtype, emphasizing the central role of metabolic abnormalities in liver damage (3). The latest epidemiological data show that the prevalence of MASLD in urban populations in China is as high as 28.77%, with approximately 16.87% of patients already exhibiting significant liver fibrosis (4). With the global prevalence of obesity and type 2 diabetes (T2DM), the risk of MASLD patients progressing to liver fibrosis, cirrhosis, and hepatocellular carcinoma has significantly increased. However, the lack of early screening methods means that most patients are diagnosed at an irreversible stage (5). Currently, liver biopsy is the gold standard for diagnosis, but due to its invasiveness, sampling errors, and procedural risks, it is difficult to widely apply in clinical practice (6). Imaging studies (such as Fibrotouch, CT, or MRI) are non-invasive but costly (7). Therefore, there is an urgent need to develop simple, cost-effective serological predictive markers.

Insulin resistance (IR) is considered the core mechanism underlying the development of MASLD (8, 9). Basic research indicates that IR promotes the influx of free fatty acids into the liver, increases lipid synthesis, and inhibits β-oxidation, leading to excessive lipid accumulation in hepatocytes (10, 11). Additionally, the chronic low-grade inflammatory state induced by IR further accelerates the activation of hepatic stellate cells and promotes fibrosis progression (12). The triglyceride-glucose index (TyG index) has been proven to be a surrogate marker for assessing IR and is closely associated with the development of MASLD (13–15). Compared with other IR surrogate markers, the TyG index exhibits high sensitivity and specificity, is easy to calculate, and has extremely low cost constraints (16). However, the traditional TyG index does not incorporate obesity as a key metabolic factor. Obesity not only exacerbates IR to promote hepatic steatosis but can also independently induce inflammation and fibrosis (17). In recent years, the TyG-BMI index (combining TyG with body mass index) has demonstrated superiority in predicting cardiovascular disease, but its value in stratifying the severity of MASLD and liver fibrosis remains controversial (18). Especially in different degrees of hepatic steatosis and fibrosis stages, there is a lack of systematic comparison of the predictive efficacy between the TyG index and the TyG-BMI index.

This study aims to address the following key questions: first, are the TyG index and TyG-BMI index independently associated with the severity of MASLD and liver fibrosis? Second, which of the two indices has superior predictive performance for hepatic steatosis and fibrosis? Additionally, is there a dose-response relationship, meaning that higher TyG/TyG-BMI levels are associated with a higher risk of liver disease?

Through a cross-sectional retrospective analysis of a large clinical dataset, this study comprehensively compared the roles of the TyG index and TyG-BMI index in MASLD risk stratification and liver fibrosis, providing more accurate and cost-effective metabolic indicator options for non-invasive screening. This helps identify high-risk populations, optimize the timing of clinical interventions, and offers new insights into the pathogenesis of MASLD.

## Research design and methods

### Study population

**Figure 1:**
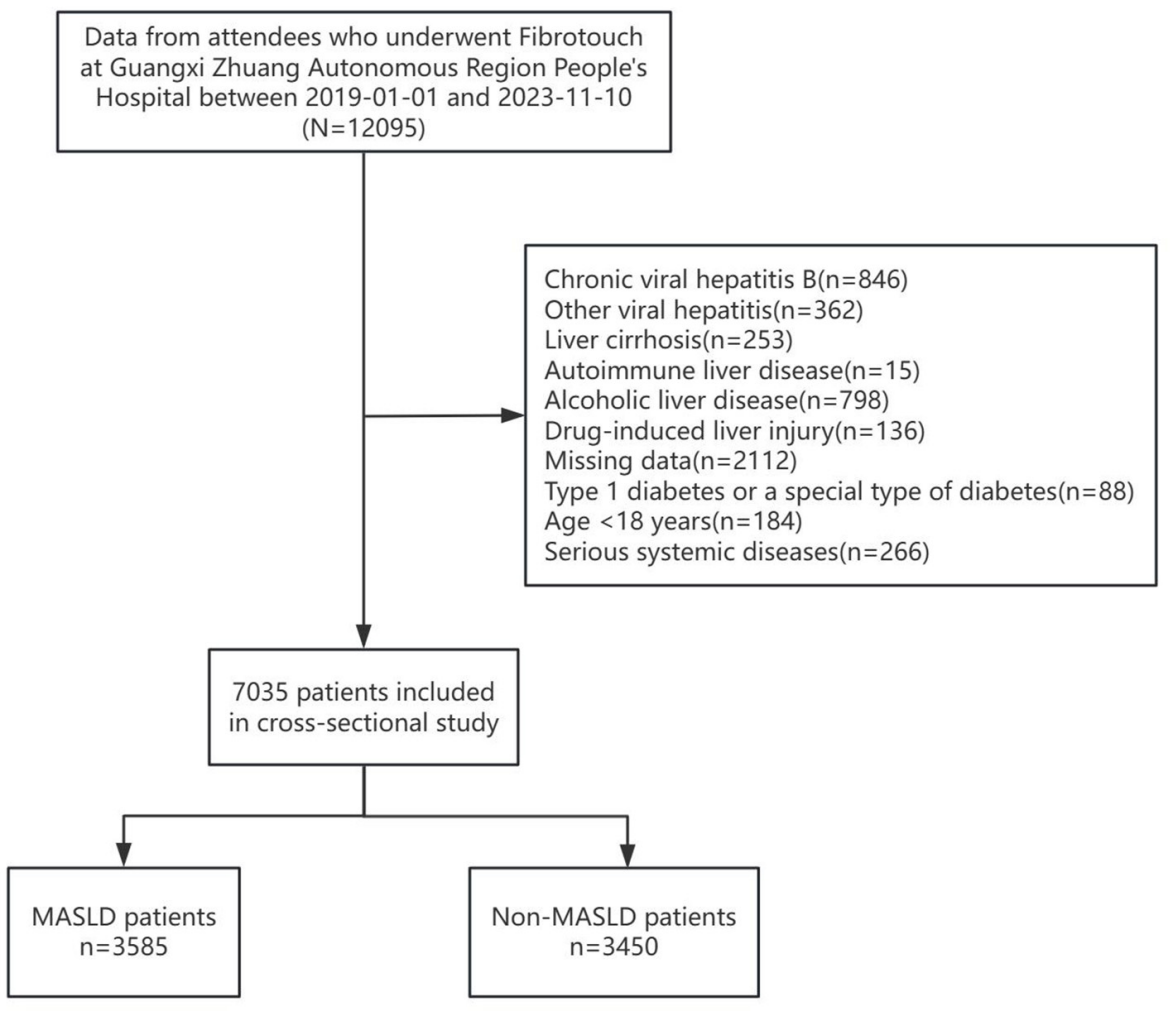
Flowchart of study participants.

This study collected data on patients who underwent Fibrotouch testing at the People's Hospital of Guangxi Zhuang Autonomous Region from January 1, 2019, to November 10, 2023. Based on the exclusion criteria, 7,035 cases were ultimately selected as the study subjects for retrospective analysis (Figure 1). Exclusion criteria included: (1) Patients with chronic viral hepatitis B, other viral hepatitis, liver cirrhosis, autoimmune liver disease, alcoholic liver disease, or drug-induced liver injury. (2) Patients with missing data. (3) Patients with type 1 diabetes or a special types of diabetes. (4) Patients under 18 years of age. (5) Patients with severe systemic diseases (including cardiovascular, pulmonary, hepatic, renal diseases, infectious diseases, mental disorders, etc.).

### Data collection

The study population's gender, age, and past medical history were obtained from outpatient medical records or admission records, and the patients' height and weight were obtained from nursing records. The laboratory biochemical indicators included: alanine transaminase (ALT), aspartate transaminase (AST), total bilirubin (TBIL), direct bilirubin (DBIL), indirect bilirubin (IBIL), alkaline phosphatase (ALP), creatinine (Cr), uric acid (UA), urea nitrogen (UREA), total cholesterol (TC), triglycerides (TG), high-density lipoprotein cholesterol (HDL-C), low-density lipoprotein cholesterol (LDL-C), and fasting blood glucose (FBG). BMI = weight/height^2^ (kg/m^2^), TyG = Ln [TG (mg/dl) × FBG (mg/dl)/2], TyG-BMI = TyG × BMI. All study participants fasted for at least 8 h after dinner, and morning fasting venous blood samples were collected for testing.

The patients were examined with hepatic transient elastography (Fibrotouch, pro5000, Wuxi Heskel Medical Technology Co., Ltd.), and they were asked to abstain from alcohol for 1 week before the examination and fast on the day of the measurement. Then, the patients were asked to assume a lying position, and an examination point was selected. Each examinee is tested an average of more than 10 times, and the median of the valid measurement values is taken to obtain the controlled attenuation parameter (CAP) and liver stiffness measurement (LSM) values, which are used to evaluate the degree of hepatic steatosis and hepatic fibrosis, respectively. All demographic data, anthropometric data, laboratory biochemical data, and inpatient medical record information were collected anonymously, with data sourced from the database of the People's Hospital of Guangxi Zhuang Autonomous Region. MASLD patients were grouped by severity based on CAP values: mild, 244 dB/m ≤ CAP < 269 dB/m; moderate, 269 dB/m ≤ CAP < 296 dB/m; severe, CAP ≥ 296 dB/m. MASLD patients were grouped based on LSM values to determine the presence of liver fibrosis: LSM ≥ 7.3 kPa was classified as the liver fibrosis group, and LSM < 7.3 kPa was classified as the non-liver fibrosis group.

## Related definitions

Definition of MASLD: (1) Imaging diagnosis of fatty liver: CAP ≥244 dB/m. (2) No other causes of hepatic steatosis or excessive alcohol consumption (≥30 g/d for males, ≥20 g/d for females). (3) At least one metabolic cardiovascular disease risk factor: ① BMI ≥25 kg/m^2^ (23 kg/m^2^ for Asia) or waist circumference (WC) >94 cm for males, >80 cm for females; ② Fasting blood glucose (FBG) ≥5.6 mmol/L (100 mg/dl) or 2-h post-load glucose levels ≥7.8 mmol/L (140 mg/dl) or glycated hemoglobin (HbA1c) ≥5.7% (39 mmol/L) or type 2 diabetes or treatment for type 2 diabetes; ③ Blood pressure ≥130/85 mmHg or specific antihypertensive drug treatment; ④ Plasma triglycerides (TG) ≥1.70 mmol/L (150 mg/dl) or lipid lowering treatment; ⑤ Plasma high-density lipoprotein cholesterol (HDL-C) ≤ 1.0 mmol/L (40 mg/dl) for males or ≤ 1.3 mmol/L (50 mg/dl) for females, or lipid lowering treatment (19). The study participants were divided into 3,450 non-MASLD cases and 3,585 MASLD cases.

The classification of MASLD based on the CAP value of Fibrotouch is as follows: a CAP value ≥ 244 dB/m is defined as hepatic steatosis; a CAP value between 244 and < 269 dB/m is defined as mild MASLD; a CAP value between 269 and < 296 dB/m is defined as moderate MASLD; and a CAP value ≥ 296 dB/m is defined as severe MASLD.

Based on the LSM value, MASLD patients are grouped according to the presence of liver fibrosis: LSM ≥ 7.3 kPa is classified as the liver fibrosis group, and LSM < 7.3 kPa is classified as the non-liver fibrosis group.

## Statistical analyses

Data analysis was performed using statistical software SPSS 26.0 (IBM Corp, Armonk, NY, USA) and R version 4.2.2 (R Foundation for Statistical Computing, Vienna, Austria). Continuous variables are expressed as mean and standard deviation. *t*-tests or analysis of variance (ANOVA) were used to compare means between two or three groups. Categorical data are presented as counts and percentages, and intergroup comparisons were performed using Pearson's chi-square test. Correlation analyses were conducted between CAP and TyG, TyG-BMI, and their components. The intergroup differences between CAP and the quartiles of TyG and TyG-BMI, as well as between TyG, TyG-BMI, and different degrees of hepatic steatosis, were analyzed by Tukey's multiple comparisons test. Logistic regression analysis was used to estimate the correlation between the TyG index, TyG-BMI index, and MASLD and liver fibrosis. Variables with *p* < 0.1 in univariate logistic regression were included in multivariate logistic regression to calculate odds ratios (OR) and 95% confidence intervals (CI). To address multicollinearity, variance inflation factors (VIF) were calculated for all variables in the initial full model. VIF > 10 was considered indicative of severe multicollinearity. Due to high multicollinearity between TC and LDL-C (VIF > 15), both TC and LDL-C were excluded. Similarly, since the TyG-BMI index is mathematically derived from TyG and BMI, BMI was excluded from models containing TyG or TyG-BMI to obtain stable estimates. After these exclusions, all variables in the final model had VIF values below 10, indicating acceptable levels of multicollinearity. Three models were established: model I (unadjusted), Model II (adjusted for age, gender, type 2 diabetes, coronary heart disease, and hypertension), and Model III (adjusted for age, gender, type 2 diabetes, coronary heart disease, hypertension, ALT, AST, TBIL, DBIL, IBIL, ALP, CR, UA, UREA, TG, HDL-C, and FBG). The predictive ability of TyG and TyG-BMI indices for MASLD and liver fibrosis was estimated using the area under the receiver operating characteristic curve (AUROC). A two-tailed value of *p* < 0.05 was considered to indicate statistical significance.

## Results

### Baseline characteristics of participants

A total of 7,035 participants (2,139 males and 4,896 females) were included in the study, among whom 3,585 (50.9%) were diagnosed with MASLD. Clinical and laboratory characteristics grouped by MASLD status are listed in Table 1. Compared with the non-MASLD group, MASLD patients had higher metabolic indices, including BMI, total cholesterol (TC), triglycerides (TG), low-density lipoprotein cholesterol (LDL-C), creatinine, uric acid, fasting blood glucose (FBG), TyG index, and TyG-BMI index, lower high-density lipoprotein cholesterol (HDL-C), and were more likely to have type 2 diabetes, coronary heart disease, and hypertension. Additionally, alanine transaminase (ALT), aspartate transaminase (AST), alkaline phosphatase (ALP), and blood urea nitrogen (UREA) were higher in MASLD patients (all *p* < 0.05).

**Table 1:** Baseline characteristics of individuals with or without MASLD.

**Variables**	**Total (*n* = 7,035)**	**Non-MASLD (*n* = 3,450)**	**MASLD (*n* = 3,585)**	***p*-value**
Age	45.1261 ± 2.035	43.2021 ± 2.147	46.9771 ± 1.632	<0.001
**Gender**				<0.001
Female	4,896 (69.595)	1,964 (56.928)	2,932 (81.785)	
Male	2,139 (30.405)	1,486 (43.072)	653 (18.215)	
**T2DM**				<0.001
No	6,185 (87.918)	3,159 (91.565)	3,026 (84.407)	
Yes	850 (12.082)	291 (8.435)	559 (15.593)	
**CHD**				<0.001
No	6,607 (93.916)	3,299 (95.623)	3,308 (92.273)	
Yes	428 (6.084)	151 (4.377)	277 (7.727)	
**Hypertension**				<0.001
No	5,857 (83.255)	3,027 (87.739)	2,830 (78.94)	
Yes	1,178 (16.745)	423 (12.261)	755 (21.06)	
BMI	24.7553 ± .457	22.5332 ± .432	26.8942 ± .905	<0.001
ALT	26.4772 ± 2.708	21.3632 ± 1.407	31.3972 ± 2.84	<0.001
AST	25.4751 ± 3.868	23.8861 ± 0.624	27.0051 ± 6.249	<0.001
TBIL	14.1185 ± .814	14.1395 ± .911	14.0985 ± .72	0.138
DBIL	2.5331 ± .478	2.5991 ± .55	2.471 ± .402	0.001
IBIL	11.6244 ± .704	11.5614 ± .719	11.6844 ± .689	0.013
ALP	71.5332 ± 1.161	68.2012 ± 0.95	74.7392 ± 0.869	<0.001
CR	80.542 ± 2.456	77.9692 ± 6.682	83.0131 ± 7.091	<0.001
UA	393.6979 ± 7.659	358.6318 ± 7.498	427.4429 ± 5.015	<0.001
UREA	4.9971 ± .275	4.8771 ± .291	5.1121 ± .249	<0.001
TC	5.3181 ± .06	5.1620 ± .999	5.4671 ± .096	<0.001
TG	1.7561 ± .706	1.260 ± .797	2.2332 ± .153	<0.001
HDL-C	1.3480 ± .29	1.4370 ± .297	1.2630 ± .254	<0.001
LDL-C	3.4580 ± .786	3.3250 ± .759	3.5860 ± .791	<0.001
FBG	5.3211 ± .198	5.0680 ± .858	5.5641 ± .41	<0.001
TyG	8.6960 ± .646	8.3980 ± .511	8.9830 ± .632	<0.001
TyG-BMI	216.1823 ± 9.497	189.662 ± 6.691	241.7043 ± 2.364	<0.001

T2DM, type 2 diabetes; CHD, coronary heart disease; BMI, body mass index; ALT, alanine transaminase;AST, aspartate transaminase;TBIL;total bilirubin;DBIL, direct bilirubin;IBIL, indirect bilirubin;ALP, alkaline phosphatase;Cr, creatinine;UA, uric acid;UREA, urea nitrogen;TC, total cholesterol;TG, triglycerides;HDL-C, high-density lipoprotein cholesterol;LDL-C, low-density lipoprotein cholesterol;FBG, fasting blood glucose.

The MASLD group was further divided into three subgroups based on CAP values: the mild MASLD group (*n* = 1,233), the moderate MASLD group (*n* = 1,511), and the severe MASLD group (*n* = 841). As shown in Table 2, the TyG index increases with the severity of hepatic steatosis (8.86 ± 0.608 vs. 8.969 ± 0.601 vs. 9.19 ± 0.671, *p* < 0.001), and the TyG-BMI index also exhibited the same trend (225.011 ± 23.916 vs. 239.351 ± 23.654 vs. 270.409 ± 37.151, *p* < 0.001).

**Table 2:** Baseline characterization of individuals based on controlled attenuation parameters (CAP) and liver stiffness measurements (LSM) in MASLD.

**Variables**	**Mild MASLD (244 ≤ CAP < 269) (*n* = 1,233)**	**Moderate MASLD (269 ≤ CAP < 296) (*n* = 1,511)**	**Severe MASLD (CAP ≥296) (*n* = 841)**	***p*-value**	**Non-liver fibrosis (LSM < 7.3) (*n* = 3,165)**	**Liver fibrosis (LSM ≥7.3) (*n* = 420)**	***p*-value**
Age	47.7631 ± 1.39	47.7051 ± 1.749	44.5181 ± 1.442	<0.001	46.7231 ± 1.388	48.8931 ± 3.182	0.001
**Gender**							
Female	965 (78.264)	1,250 (82.727)	717 (85.256)	<0.001	2,568 (81.137)	364 (86.667)	0.006
Male	268 (21.736)	261 (17.273)	124 (14.744)		597 (18.863)	56 (13.333)	
**T2DM**							
No	1,042 (84.509)	1,277 (84.514)	707 (84.067)	0.953	2,697 (85.213)	329 (78.333)	<0.001
Yes	191 (15.491)	234 (15.486)	134 (15.933)		468 (14.787)	91 (21.667)	
**CHD**							
No	1,140 (92.457)	1,393 (92.191)	775 (92.152)	0.956	2,931 (92.607)	377 (89.762)	0.04
Yes	93 (7.543)	118 (7.809)	66 (7.848)		234 (7.393)	43 (10.238)	
**Hypertension**							
No	962 (78.021)	1,191 (78.822)	677 (80.499)	0.393	2,522 (79.684)	308 (73.333)	0.003
Yes	271 (21.979)	320 (21.178)	164 (19.501)		643 (20.316)	112 (26.667)	
BMI	25.4092 ± .183	26.6962 ± .061	29.4253 ± .412	<0.001	26.592 ± .564	29.1794 ± .071	<0.001
ALT	26.592 ± 1.04	30.462 ± 0.137	40.132 ± 7.125	<0.001	29.6932 ± 0.599	44.2423 ± 2.722	<0.001
AST	26.0832 ± 2.525	26.2611 ± 0.122	29.6921 ± 3.738	<0.001	25.9181 ± 1.298	35.1973 ± 4.905	<0.001
TBIL	14.2126 ± .455	14.2355 ± .456	13.6844 ± .977	0.056	14.0745 ± .827	14.2744 ± .844	0.441
DBIL	2.5031 ± .986	2.491 ± .016	2.3850 ± .858	0.128	2.4541 ± .454	2.5890 ± .908	0.009
IBIL	11.8674 ± .998	11.7134 ± .609	11.3624 ± .342	0.053	11.6754 ± .707	11.7474 ± .556	0.763
ALP	74.0791 ± 9.466	74.1521 ± 9.803	76.7622 ± 4.334	0.006	74.171 ± 9.581	79.0312 ± 8.444	<0.001
CR	82.931 ± 7.276	83.551 ± 7.209	82.1721 ± 6.585	0.169	82.841 ± 6.601	84.3182 ± 0.386	0.155
UA	410.0038 ± 9.396	426.5519 ± 1.916	454.6121 ± 02.098	<0.001	425.2599 ± 3.074	443.8921 ± 07.234	<0.001
UREA	5.151 ± .27	5.1061 ± .279	5.0681 ± .158	0.328	5.1171 ± .227	5.081 ± .404	0.607
TC	5.4781 ± .104	5.421 ± .058	5.5341 ± .148	0.049	5.4731 ± .085	5.4241 ± .173	0.422
TG	2.0222 ± .128	2.1261 ± .522	2.7332 ± .939	<0.001	2.1911 ± .973	2.5493 ± .185	0.025
HDL-C	1.3060 ± .268	1.2610 ± .251	1.2030 ± .228	<0.001	1.2670 ± .255	1.2320 ± .251	0.007
LDL-C	3.5880 ± .783	3.5630 ± .78	3.6260 ± .82	0.174	3.5940 ± .778	3.5260 ± .882	0.131
FBG	5.411 ± .262	5.5721 ± .44	5.7751 ± .531	<0.001	5.5061 ± .353	6.0051 ± .719	<0.001
TyG	8.860 ± .608	8.9690 ± .601	9.190 ± .671	<0.001	8.9640 ± .618	9.1250 ± .715	<0.001
TyG-BMI	225.0112 ± 3.916	239.3512 ± 3.654	270.4093 ± 7.151	<0.001	238.4062 ± 8.781	266.5584 ± 4.753	<0.001

T2DM, type 2 diabetes; CHD, coronary heart disease; BMI, body mass index;ALT, alanine transaminase; AST, aspartate transaminase; TBIL, total bilirubin; DBIL, direct bilirubin; IBIL, indirect bilirubin;ALP, alkaline phosphatase; Cr, creatinine; UA, uric acid; UREA, urea nitrogen; TC, total cholesterol; TG, triglycerides; HDL-C, high-density lipoprotein cholesterol; LDL-C, low-density lipoprotein cholesterol; FBG, fasting blood glucose.

Among MASLD patients, 420 had liver fibrosis, and 3,165 had non-liver fibrosis. Compared with non-liver fibrosis patients, liver fibrosis patients were older and had higher BMI, ALT, AST, ALP, triglycerides, uric acid, FBG, TyG index, and TyG-BMI index, as well as lower HDL-C (all *p* < 0.05; Table 2).

### Comparison of TyG and TyG-BMI among different degrees of hepatic steatosis

**Figure 2:**
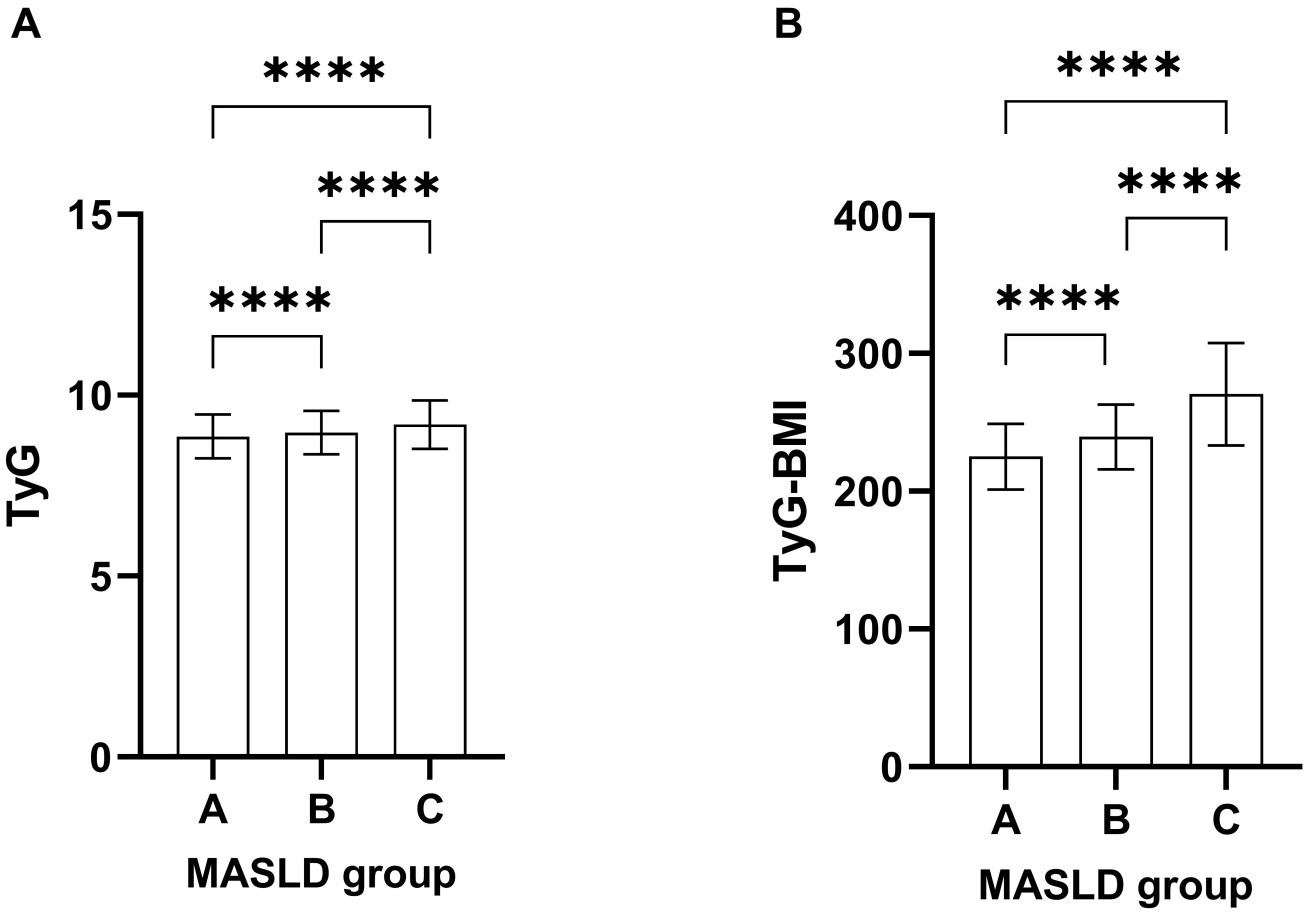
Comparison between the TyG **(A)** and TyG-BMI **(B)** index among different degrees of hepatic steatosis. The error bars represent standard deviation (SD) A: mild MASLD group; B: moderate MASLD group; C: severe MASLD group; ****p < 0.0001.

Based on the degree of hepatic steatosis, the subjects were divided into three groups: mild (*n* = 1,233), moderate (*n* = 1,511), and severe (*n* = 841). The TyG index and TyG-BMI index showed statistically significant differences among all groups (Figures 2A, B).

### Comparison of CAP between the TyG and TyG-BMI quartiles

**Figure 3:**
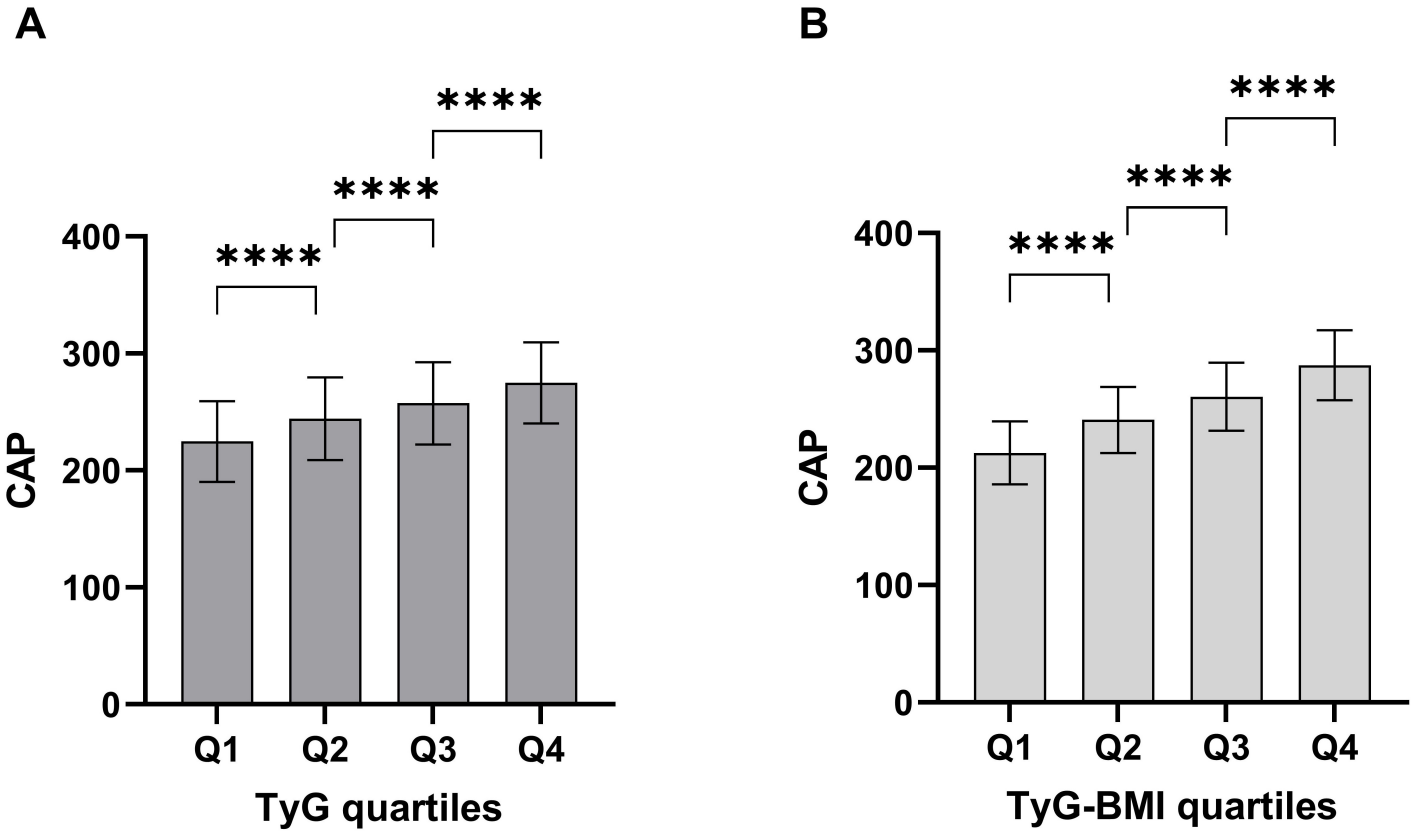
Comparison of CAP in the study population between the TyG **(A)** and TyG-BMI **(B)** quartiles. The error bars represent standard deviation (SD) classification of TyG quartiles: Q1 (−8.233), Q2 (8.234–8.635), Q3 (8.636–9.073), Q4 (9.074–); TyG-BMI quartiles: Q1 (−188.868), Q2 (188.869–213.887), Q3 (213.888–240.035), Q4 (240.036–) ****p < 0.0001.

The study subjects were divided into four groups based on the TyG index and TyG-BMI index. CAP values gradually increased with increasing TyG index quartiles (all *p* < 0.001; Figure 3A) and TyG-BMI index quartiles (all *p* < 0.001; Figure 3B).

### Correlation analysis between CAP and TyG, TyG-BMI, and their components

**Figure 4:**
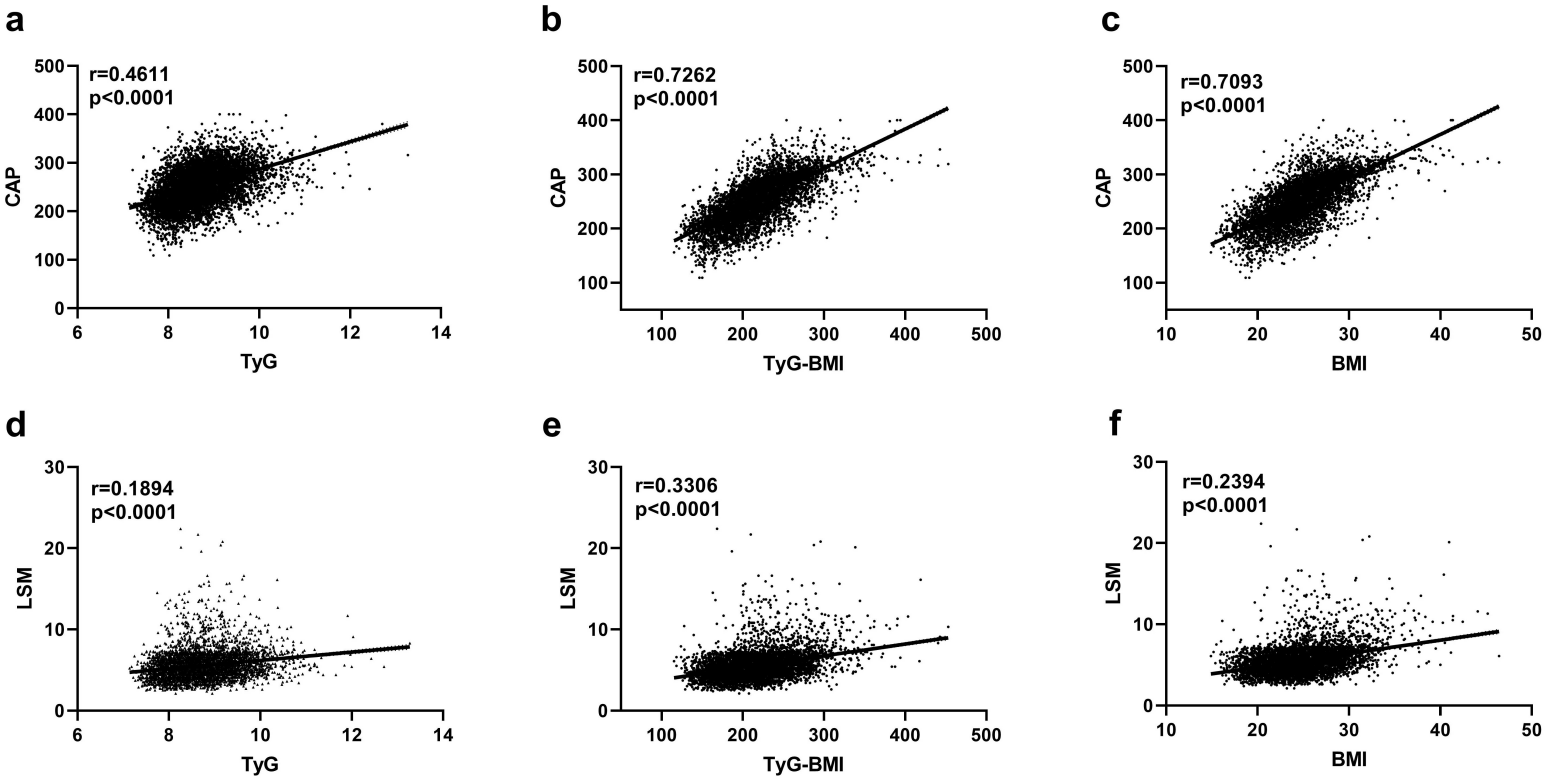
Univariate correlation analysis between CAP and TyG **(a)**, TyG-BMI **(b)**, and BMI **(c)**, as well as between LSM and TyG **(d)**, TyG-BMI **(e)**, and BMI **(f)**. CAP, controlled attenuation parameter; LSM, liver stiffness measurement; TyG, triglyceride glucose index; TyG-BMI, triglyceride glucose-body mass index; BMI, body mass index.

Correlation analysis showed that CAP was a positive association with TyG, TyG-BMI, and BMI, with the highest correlation coefficient between CAP and TyG-BMI. LSM was also a positive association with TyG, TyG-BMI, and BMI, with the highest correlation coefficient between LSM and TyG-BMI (Figure 4).

### The TyG, TyG-BMI index is associated with hepatic steatosis severity and liver fibrosis

Multivariate logistic regression analysis revealed that both the TyG index and TyG-BMI index were significantly positively correlated with the severity of MASLD (Table 3). Due to severe multicollinearity between TC and LDL-C [variance inflation factor (VIF) >10], and severe multicollinearity between BMI, TyG, and TyG-BMI, after excluding BMI, the VIF values for TyG and TyG-BMI were both less than 10. Therefore, TC, LDL, and BMI were excluded when constructing the regression model. In Model I, which did not adjust for any covariates, the OR values for the TyG index in relation to mild, moderate, and severe MASLD were 4.974 (95% CI: 4.373–5.658), 6.659 (95% CI: 5.879–7.542), and 10.926 (95% CI: 9.440–12.645), respectively, with all *p*-values < 0.001. After adjusting for age, gender, type 2 diabetes, coronary heart disease, hypertension, ALT, AST, TBIL, DBIL, IBIL, ALP, CR, UA, UREA, TG, HDL-C, and FBG variables (Model III), these associations remained highly significant despite some attenuation (OR values of 3.386, 4.522, and 5.701, respectively). Notably, the TyG-BMI index showed a stronger association. In Model III, for each 1-unit increase in the TyG-BMI index, the risk of mild, moderate, and severe MASLD increased by 5.9% (OR = 1.059, 95% CI: 1.056–1.063), 8.8% (OR = 1.088, 95% CI: 1.084–1.092), and 13.1% (OR = 1.131, 95% CI: 1.126–1.136), respectively.

**Table 3:** Logistic regression was used to explore the relationship between TyG index, TyG-BMI index and the severity of hepatic steatosis and liver fibrosis.

**Variables**	**TyG index**			**TyG-BMI index**		
	**Model I**	**Model II**	**Model III**	**Model I**	**Model II**	**Model III**
	**OR (95%CI)**	**OR (95%CI)**	**OR (95%CI)**	**OR (95%CI)**	**OR (95%CI)**	**OR (95%CI)**
Non-MASLD	Reference	Reference	Reference	Reference	Reference	Reference
Mild MASLD	4.974 (4.373, 5.658)	4.032 (3.531, 4.605)	3.386 (3.075, 3.728)	1.059 (1.055, 1.062)	1.058 (1.054, 1.062)	1.059 (1.056, 1.063)
*p*-value	<0.001	<0.001	<0.001	<0.001	<0.001	<0.001
Moderate MASLD	6.659 (5.879, 7.542)	5.324 (4.684, 6.051)	4.522 (4.097, 4.992)	1.082 (1.078, 1.087)	1.082 (1.078, 1.086)	1.088 (1.084, 1.092)
*p*-value	<0.001	<0.001	<0.001	<0.001	<0.001	<0.001
Severe MASLD	10.926 (9.440, 12.645)	9.164 (7.892, 10.640)	5.701 (5.070, 6.411)	1.120 (1.115, 1.126)	1.119 (1.113, 1.124)	1.131 (1.126, 1.136)
*p*-value	<0.001	<0.001	<0.001	<0.001	<0.001	<0.001
Non-liver fibrosis	Reference	Reference	Reference	Reference	Reference	Reference
Liver fibrosis	1.455 (1.251, 1.693)	1.420 (1.218, 1.655)	0.903 (0.670, 1.217)	1.023 (1.020, 1.026)	1.025 (1.022, 1.029)	1.029 (1.025, 1.033)
*p*-value	<0.001	<0.001	0.504	<0.001	<0.001	<0.001

Model I: unadjusted.

Model II: adjusted for age, gender, type 2 diabetes, coronary heart disease, and hypertension.

Model III: adjusted for age, gender, type 2 diabetes, coronary heart disease, hypertension, ALT, AST, TBIL, DBIL, IBIL, ALP, CR, UA, UREA, TG, HDL-C, and FBG.

ALT, alanine transaminase;AST, aspartate transaminase;TBIL;total bilirubin;DBIL, direct bilirubin;IBIL, indirect bilirubin;ALP, alkaline phosphatase;Cr, creatinine;UA, uric acid;UREA, urea nitrogen;TG, triglycerides;HDL-C, high-density lipoprotein cholesterol;FBG, fasting blood glucose.

In the analysis of liver fibrosis, the TyG index was significantly associated with liver fibrosis in the unadjusted model (Model I; OR = 1.455, 95% CI: 1.251–1.693, *p* < 0.001), but this association disappeared in the fully adjusted model (Model III; OR = 0.903, 95% CI: 0.670–1.217, *p* = 0.504). In contrast, the TyG-BMI index remained significantly associated in all models, suggesting that TyG-BMI is an independent predictor of liver fibrosis. Even after full adjustment, each additional unit of TyG-BMI was associated with a 2.9% increase in the risk of liver fibrosis (OR = 1.029, 95% CI: 1.025–1.033, *p* < 0.001).

The quartile analysis in Table 4 further validated the aforementioned findings. For hepatic steatosis, after adjusting for all confounding factors (Model III), the TyG index (OR = 4.083, 95% CI: 3.446–4.838, *p* < 0.001) and TyG-BMI index (OR = 1.076, 95% CI: 1.071–1.080, *p* < 0.001) were significantly associated with MASLD. The risk in the highest quartile (Q4) of the TyG index was 4.482 times that of the lowest quartile (Q1; 95% CI: 3.348–6.001, *p* < 0.001), while the risk in the TyG-BMI index Q4 group was as high as 239.410 times that of the Q1 group (95% CI: 170.987–335.214, *p* < 0.001). This dose-response relationship was highly significant in trend tests (*p*-trend < 0.001), indicating that the higher the TyG and TyG-BMI levels, the greater the risk of developing MASLD. In the analysis of liver fibrosis, the TyG index was not significantly associated with liver fibrosis (OR = 0.903, 95% CI: 0.670–1.217, *P* = 0.504), and no significant differences were observed between the quartile groups of the TyG index (Q4 vs. Q1: OR = 0.894, 95% CI: 0.618–1.293, *p* = 0.551). However, the TyG-BMI index was significantly associated with liver fibrosis (OR = 1.029, 95% CI: 1.025–1.033, *p* < 0.001), with the risk of liver fibrosis in the Q4 group being 4.585 times higher than in the Q1 group (95% CI: 3.211–6.486, *p* < 0.001), and a significant trend was observed (*p*-trend < 0.001). In Models I, II, and III, the trend *p*-values were all < 0.05, indicating that the higher the TyG-BMI index level, the higher the risk of liver fibrosis.

**Table 4:** Logistic analysis according to TyG, TyG-BMI quartiles associated with MASLD and liver fibrosis.

	**MASLD**						**Liver fibrosis**					
**Variables**	**Model I**		**Model II**		**Model III**		**Model I**		**Model II**		**Model III**	
	**OR (95%CI)**	***p*** **-value**	**OR(95%CI)**	***p*** **-value**	**OR (95%CI)**	***p*** **-value**	**OR(95%CI)**	***p*** **-value**	**OR (95%CI)**	***p*** **-value**	**OR (95%CI)**	***p*** **-value**
TyG index	6.613 (5.937, 7.365)	<0.001	5.398 (4.831, 6.031)	<0.001	4.083 (3.446, 4.838)	<0.001	1.455 (1.251, 1.693)	<0.001	1.372 (1.173, 1.605)	<0.001	0.903 (0.670, 1.217)	0.504
**TyG (quartile)**												
Q1	Reference		Reference		Reference		Reference		Reference		Reference	
Q2	2.896 (2.491, 3.368)	<0.001	2.368 (2.027, 2.768)	<0.001	1.761 (1.490, 2.082)	<0.001	1.054 (0.777, 1.429)	0.737	1.018 (0.749, 1.384)	0.908	0.867 (0.629, 1.195)	0.384
Q3	6.156 (5.291, 7.162)	<0.001	4.742 (4.054, 5.547)	<0.001	2.602 (2.155, 3.141)	<0.001	1.027 (0.756, 1.396)	0.863	0.988 (0.725, 1.345)	0.937	0.727 (0.521, 1.014)	0.060
Q4	18.946 (15.989, 22.450)	<0.001	13.558 (11.373, 16.162)	<0.001	4.482 (3.348, 6.001)	<0.001	1.719 (1.297, 2.278)	<0.001	1.552 (1.164, 2.071)	0.003	0.894 (0.618, 1.293)	0.551
*p* for trend	<0.001		<0.001		<0.001		<0.001		0.003		0.361	
**TyG- BMI index**	1.073 (1.069, 1.076)	<0.001	1.073 (1.069, 1.076)	<0.001	1.076 (1.071, 1.080)	<0.001	1.023 (1.020, 1.026)	<0.001	1.025 (1.022, 1.029)	<0.001	1.029 (1.025, 1.033)	<0.001
**TyG-BMI (quartile)**												
Q1	Reference		Reference		Reference		Reference		Reference		Reference	
Q2	12.854 (9.879, 16.727)	<0.001	12.213 (9.374, 15.912)	<0.001	10.565 (8.056, 13.857)	<0.001	1.078 (0.740, 1.571)	0.696	1.071 (0.733, 1.564)	0.725	1.051 (0.712, 1.553)	0.801
Q3	68.859 (52.783, 89.832)	<0.001	64.923 (49.694, 84.820)	<0.001	51.525 (38.885, 68.273)	<0.001	1.656 (1.169, 2.346)	0.004	1.648 (1.159, 2.343)	0.005	1.650 (1.145, 2.379)	0.007
Q4	341.864 (251.735, 464.263)	<0.001	325.472 (239.505, 442.296)	<0.001	239.410 (170.987, 335.214)	<0.001	4.741 (3.474, 6.470)	<0.001	4.983 (3.619, 6.861)	<0.001	4.563 (3.211, 6.486)	<0.001
*p* for trend	<0.001		<0.001		<0.001		<0.001		<0.001		<0.001	

Model I: unadjusted.

Model II: adjusted for age, gender, type 2 diabetes, coronary heart disease, and hypertension.

Model III: adjusted for age, gender, type 2 diabetes, coronary heart disease, hypertension, ALT, AST, TBIL, DBIL, IBIL, ALP, CR, UA, UREA, TG, HDL-C, and FBG.

ALT, alanine transaminase;AST, aspartate transaminase;TBIL;total bilirubin;DBIL, direct bilirubin;IBIL, indirect bilirubin;ALP, alkaline phosphatase;Cr, creatinine;UA, uric acid;UREA, urea nitrogen;TG, triglycerides;HDL-C, high-density lipoprotein cholesterol;FBG, fasting blood glucose.

Restricted cubic spline analysis revealed a J-shaped non-linear relationship between TyG-BMI and MASLD risk (Supplementary Figure S1). The association strength increased sharply when TyG-BMI exceeded approximately 213.887. Furthermore, subgroup analysis demonstrated that the TyG-BMI index maintained highly significant predictive capability for MASLD risk across all subgroups (all *p* < 0.001). Interaction analysis revealed significant heterogeneity in the strength of this association. Among individuals aged < 50 years, the effect of TyG-BMI was markedly stronger than in those aged ≥50 years (*p* for interaction = 0.004); similarly, the effect was significantly stronger in non-diabetic individuals than in diabetic individuals (*p* for interaction < 0.001). A similar pattern of heterogeneity was observed for the TyG index (*p* for interaction values < 0.001 and < 0.001, respectively; Table S1). For liver fibrosis risk, the TyG-BMI index remained significantly associated across all subgroups (all *p* < 0.001), with stronger associations in men than women (*p* for interaction = 0.038) and a trend toward greater strength in younger individuals (< 50 years; *p* for interaction = 0.031). In contrast, the association between the TyG index and liver fibrosis did not reach statistical significance in any subgroup (all *p* > 0.05; Table S2).

### Predictive ability of TyG and TyG-BMI for MASLD and liver fibrosis

**Figure 5:**
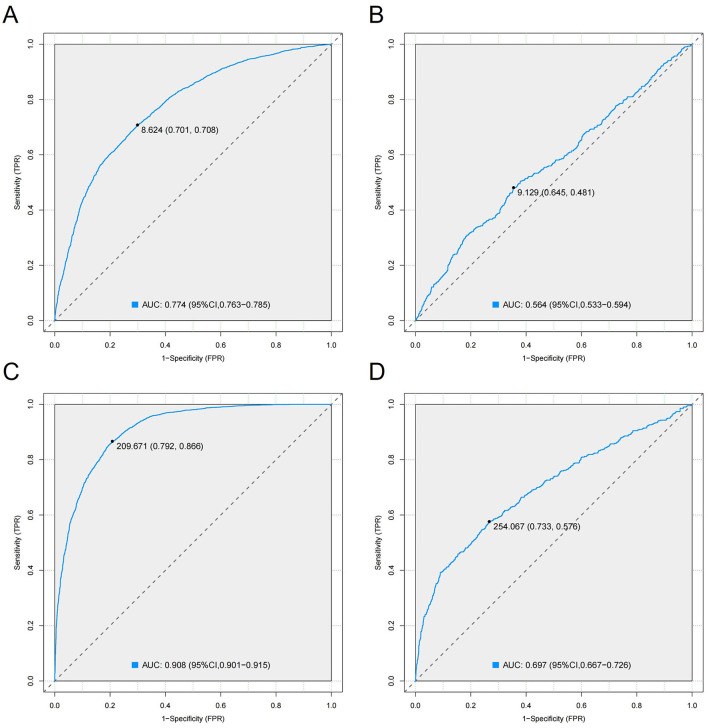
ROC curve analysis for evaluating the predictive ability of TyG and TyG-BMI for MASLD and liver fibrosis. **(A)** ROC curve for TyG index predicting MASLD; **(B)** ROC curve for TyG index predicting liver fibrosis; **(C)** ROC curve for TyG-BMI index predicting MASLD; **(D)** ROC curve for TyG-BMI index predicting liver fibrosis.

ROC curve analysis was used to evaluate the predictive ability of TyG and TyG-BMI for MASLD and liver fibrosis (Figure 5). The AUC value for the TyG index in predicting MASLD was 0.774 (95% CI 0.763–0.785), with a cutoff value of 8.624, specificity of 70.1%, and sensitivity of 70.8%. In other words, if the TyG index is 8.624 or higher, it can be used as a predictive indicator for MASLD (Figure 5A). The AUC value of the TyG index for liver fibrosis (AUC = 0.564, 95% CI 0.533–0.594) was relatively lower than that for MASLD. In this case, the TyG index is not reliable as a predictive indicator for liver fibrosis in MASLD patients (Figure 5B). The AUC value for the TyG-BMI index in predicting MASLD was 0.908 (95% CI 0.901–0.915), with a cutoff value of 209.671, specificity of 79.2%, and sensitivity of 86.7% (Figure 5C). The AUC value of the TyG-BMI index for predicting liver fibrosis was 0.697 (95% CI 0.667–0.726), with a cutoff value of 254.067, specificity of 73.3%, and sensitivity of 57.6% (Figure 5D). Therefore, when the TyG-BMI index is 209.671 or higher, it can serve as a predictive indicator for MASLD; when the TyG-BMI index exceeds 254.067, MASLD patients have a higher risk of developing liver fibrosis. The results indicate that the TyG-BMI index significantly outperforms the TyG index in predicting MASLD (AUC: 0.908 vs. 0.774) and also has a higher predictive ability for liver fibrosis (AUC: 0.697 vs. 0.564).

## Discussion

In recent years, with the rapid development of China's economy and changes in lifestyle, the incidence of MASLD has shown a significant upward trend. This disease is characterized by high genetic susceptibility, an increasing proportion of young patients, insufficient public awareness, and limited clinical diagnostic and treatment options (20). Notably, MASLD not only leads to end-stage liver diseases such as decompensated cirrhosis and hepatocellular carcinoma but also increases the risk of type 2 diabetes, cardiovascular disease, chronic kidney disease, and non-hepatic malignant tumors, imposing a significant burden on global public health systems (21–23). Due to the lack of obvious symptoms and slow progression in the early stages of MASLD, patients often underestimate its severity. However, clinical observations have revealed that many patients diagnosed incidentally with hepatic steatosis actually already exhibit significant risks of liver fibrosis (24). This situation underscores the importance and urgency of developing accurate, convenient, and non-invasive diagnostic tools.

Early risk assessment and intervention in the management of MASLD not only prevent liver-related complications but also reduce the risk of metabolic abnormalities such as type 2 diabetes and cardiovascular disease. Among numerous biomarkers, the TyG index has garnered significant attention as a reliable surrogate marker for insulin resistance (13, 25). However, current research on the correlation between the TyG index and the progression of liver fibrosis and the severity of steatosis in MASLD remains insufficient, particularly regarding the predictive value of the TyG index for the risk of liver fibrosis in MASLD patients. Therefore, further investigating the relationship between TyG and its derived indicator (TyG-BMI) with liver fat content and fibrosis severity, and evaluating their predictive efficacy for MASLD, will provide new theoretical basis and clinical strategies for the early screening and intervention of this disease.

This study systematically compared the predictive value of the TyG index and TyG-BMI index for different severities of MASLD and liver fibrosis. The results showed that both TyG and TyG-BMI were independently associated with the severity of MASLD, but TyG-BMI demonstrated superior predictive performance (AUC 0.908 vs. 0.774). After adjusting for multiple confounding factors, only TyG-BMI remained significantly associated with liver fibrosis. Both indices exhibited a clear dose-response relationship, with the highest quartile of TyG-BMI associated with a 239-fold increased risk of hepatic steatosis. Additionally, potential cutoff values for clinical risk stratification were identified. These findings provide new evidence for understanding the metabolic drivers of MASLD and reveal the synergistic role of obesity and insulin resistance in liver disease progression.

This study confirmed that the TyG index is significantly associated with the severity of MASLD, a finding that is highly consistent with the results of Zhang et al.'s (26) large-scale cohort study. Their study showed that for every 1-unit increase in the TyG index, the risk of MASLD increased by 78.4% (95% CI = 1.383–2.302, *p* < 0.001), while in this study, the risk in the highest quartile of the TyG index increased to 4.48 times. This difference may be attributed to the stricter liver imaging assessment criteria used in this study. Furthermore, our study further refined the dose-response relationship between the TyG index and different degrees of fatty degeneration (mild, moderate, and severe), which has been rarely reported in previous studies. Additionally, the excellent predictive performance of the TyG-BMI index is consistent with recent studies, indicating that composite metabolic indicators are superior to single biomarkers (27–30). Our results are consistent with those of Kim et al. (31), who reported that the TyG-BMI index achieved an AUC of 0.862–0.872 and a sensitivity of 86.3% in predicting MASLD in the general population. From a mechanistic perspective, this advantage may stem from TyG-BMI being a combination of TyG and BMI levels, thereby reflecting both insulin resistance and obesity severity. Obesity not only exacerbates hepatic lipid accumulation through insulin resistance-dependent pathways (increased free fatty acid influx) but also promotes disease progression via insulin resistance-independent mechanisms (e.g., dysregulation of adipokines) (32). Notably, the risk of severe steatosis in the TyG-BMI Q4 group (OR = 239.41) was significantly higher than that in the TyG Q4 group (OR = 4.48), suggesting that BMI may amplify the metabolic damage caused by insulin resistance. This finding supports the “multiple hit” hypothesis for the onset of MASLD (8).

Through restricted cubic spline analysis, we identified a significant “J-shaped” non-linear association between TyG-BMI and MASLD risk. The inflection point occurred at approximately TyG-BMI 213, with risk surging sharply beyond this threshold. This confirms that the ultra-high quartile OR value reflects a genuine dose-response inflection point, suggesting TyG-BMI > 213 serves as a critical warning threshold for metabolic decompensation and steeply increasing risk. Furthermore, subgroup analyses revealed significant population differences. TyG-BMI demonstrated the strongest predictive power for MASLD in younger (< 50 years) and non-diabetic populations, highlighting its exceptional value for early screening of metabolic diseases, particularly in contemporary populations experiencing increasingly younger onset. Furthermore, its association with liver fibrosis was stronger in males. These findings suggest that future clinical applications may require establishing refined risk assessment criteria based on demographic characteristics.

Our study found significant differences between the two indices in predicting liver fibrosis: the TyG index lost its significance after adjusting for multiple confounding factors (OR = 0.903, *p* = 0.504), while TyG-BMI remained strongly correlated (OR = 1.029, *p* < 0.001). This difference may reflect that obesity-related pathways (such as chronic inflammation and gut microbiota dysbiosis) may independently drive fibrosis beyond insulin resistance. Visceral fat-released pro-fibrotic cytokines (TGF-β, IL-17) can directly activate hepatic stellate cells (33). The association between TyG and liver fibrosis weakened after adjusting for variables such as ALT and AST, suggesting that the correlation may be mediated by hepatocyte damage rather than insulin resistance alone. Interestingly, these findings contrast with those from cardiovascular studies, which show that TyG alone can robustly predict cardiovascular disease outcomes (34–36), suggesting that different organs have distinct mechanisms of response to metabolic abnormalities.

The TyG index primarily reflects insulin resistance in peripheral tissues, specifically the imbalance in glucose and free fatty acid metabolism. However, the TyG index alone may inadequately assess the dysfunction of adipose tissue itself, which is a key driver of metabolic inflammation and liver injury (37, 38). The TyG-BMI index quantifies overall obesity burden by integrating BMI. This integration is mechanistically crucial. First, enlarged adipose tissue—particularly visceral fat—serves as a primary source of pro-inflammatory cytokines (e.g., TNF-α, IL-6) and lipotoxic substances (e.g., ceramides). These factors directly impact the liver via the portal venous system, inducing and amplifying hepatic inflammation and fibrosis (39). Second, obesity itself induces adipocyte hypoxia, endoplasmic reticulum stress, and macrophage infiltration, establishing a chronic low-grade inflammatory state independent of classical peripheral insulin resistance pathways (40). Therefore, TyG-BMI may simultaneously quantify the dual hepatic impact of metabolic dysfunction (TyG) and inflammatory burden (BMI), potentially representing the core mechanism underlying its superior predictive value for liver fibrosis compared to TyG alone.

To comprehensively evaluate the clinical value of TyG-BMI, we compared it with other commonly used non-invasive indicators. While HOMA-IR, the “gold standard” for insulin resistance, is more direct, it relies on insulin testing, which is costly and exhibits significant inter-laboratory variability, making it unsuitable for large-scale screening (16). In contrast, TyG and TyG-BMI require only routine lipid and glucose data, offering greater universality. Compared to other composite formulas like the Fatty Liver Index (FLI) and Visceral Adipose Index (VAI), TyG-BMI demonstrates distinct advantages. A recent comparative study revealed that in predicting MAFLD in the Chinese population, TyG-BMI (AUC = 0.903) showed significantly superior discriminatory performance compared to FLI (AUC = 0.879) and VAI (AUC = 0.773) (41). This may be because FLI focuses more on hepatic cell injury and central obesity, while VAI primarily estimates visceral fat function, and TyG-BMI more directly integrates the core metabolic axis driving fatty liver—insulin resistance and overall obesity.

TyG-BMI can serve as a first-line screening tool for high-risk populations for MASLD (e.g., central obesity, diabetes), particularly in resource-limited settings such as community hospitals. In this study, TyG-BMI demonstrated predictive ability for fibrosis (AUC = 0.697) comparable to that reported for the liver fibrosis index (FIB-4) by Sun et al. (42), but TyG-BMI is simpler to calculate and thus more suitable for primary care screening. It is important to note that while TyG-BMI has some predictive ability for liver fibrosis in MASLD patients, its AUC remains lower than that of elastography (AUC ≈ 0.85). Therefore, positive cases require further imaging confirmation (43).

This study had a large sample size and employed standardized data collection methods to measure and evaluate participants' various indicators, while controlling for most potential confounding factors (including liver function indicators and multiple metabolic parameters), which enhances the reliability of our findings. However, the limitations of our study should not be overlooked. First, the cross-sectional design cannot establish causal relationships, and prospective studies are needed to validate the predictive value of TyG-BMI for fibrosis progression. Second, the absence of histological confirmation may underestimate the prevalence of liver fibrosis. Furthermore, unaccounted confounding factors (such as diet and exercise) may influence the results. Finally, the study population originated from a single center in China, potentially limiting the generalizability of our findings to other geographic regions and ethnic groups worldwide. Metabolic characteristics, body composition, and the prevalence of MASLD drivers may vary significantly across different regions and ethnic populations. Additionally, we employed FibroTouch as the primary assessment tool for hepatic steatosis and fibrosis. Although this technology is widely used and validated in China, its diagnostic thresholds—particularly for staging liver fibrosis—may be influenced by ethnicity, etiology, and device model. Future longitudinal studies are needed to validate the predictive value of TyG-BMI for liver disease progression. If feasible, comparisons of the predictive efficacy of TyG-BMI vs. emerging biomarkers (such as FIB-4, PRO-C3) across different racial populations should be conducted, and the potential of TyG-BMI-guided interventions (such as bariatric surgery) to improve liver disease outcomes should be explored.

## Conclusion

The TyG-BMI index is a strong predictor of the severity of MASLD and liver fibrosis, with predictive performance superior to that of the traditional TyG index, making it an effective tool for clinical screening of high-risk populations for MASLD. As an indicator combining superiority and clinical practicality, TyG-BMI can effectively stratify MASLD risk while reflecting insulin resistance and obesity-related metabolic abnormalities. Its strong correlation with liver fibrosis underscores the importance of addressing obesity in MASLD management. Although further validation is needed, TyG-BMI holds promise for revolutionizing the screening paradigm for metabolic liver diseases.

## Data Availability

The raw data supporting the conclusions of this article will be made available by the authors, without undue reservation.
