# Supplementary figures and images for: The relationship between triglyceride-glucose index, triglyceride-glucose-body mass index, and the severity of hepatic steatosis and liver fibrosis in patients with MASLD: a cross-sectional study
Source: Front Nutr. 2026 Feb 26;13:1740308. doi: 10.3389/fnut.2026.1740308 (PMC12979549; doi:10.3389/fnut.2026.1740308)

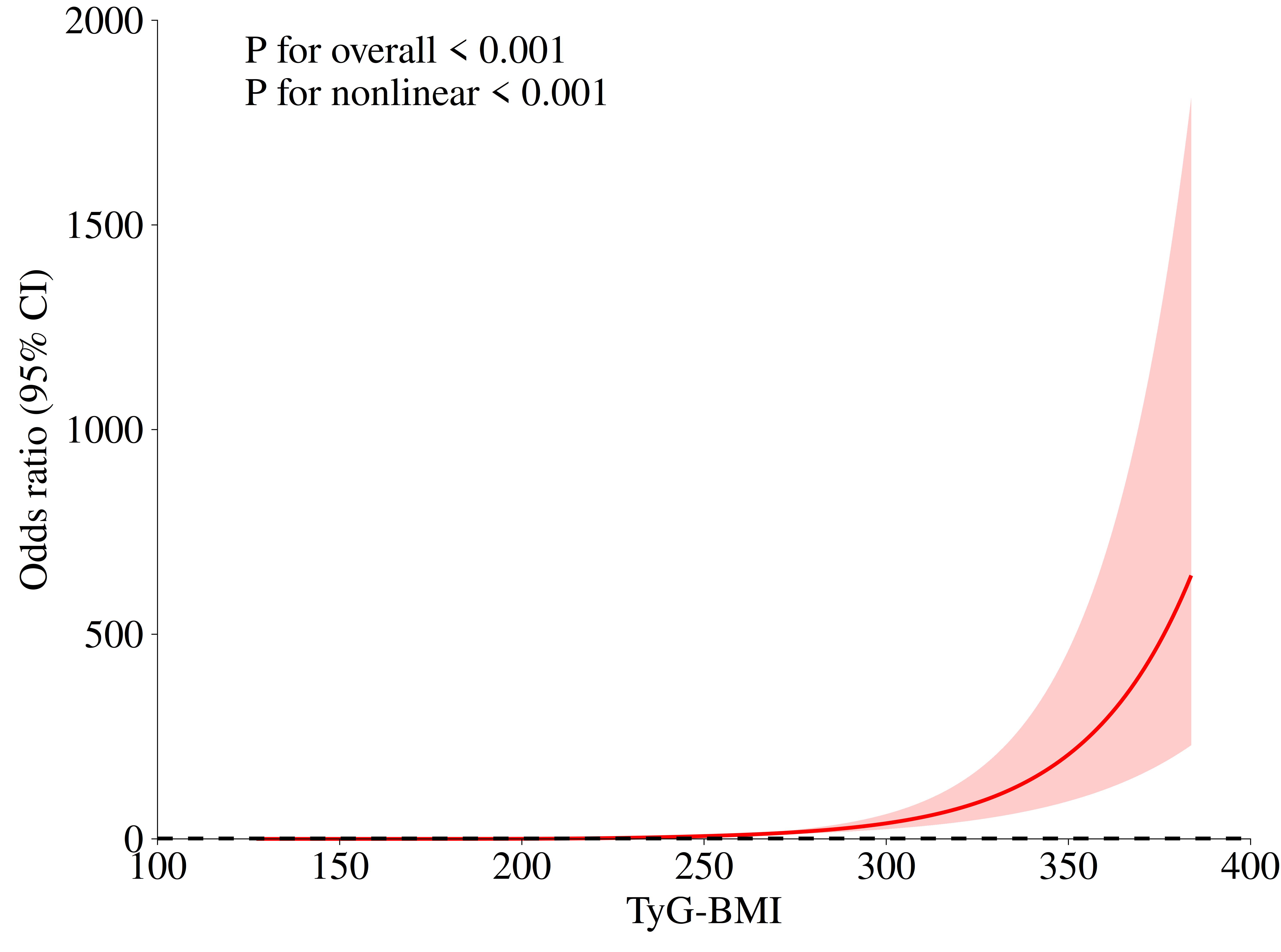

Supplement: Supplementary file 1 [file Image_1.jpeg]
