# Supplementary material for: The relationship between triglyceride-glucose index, triglyceride-glucose-body mass index, and the severity of hepatic steatosis and liver fibrosis in patients with MASLD: a cross-sectional study
Source: Front Nutr. 2026 Feb 26;13:1740308. doi: 10.3389/fnut.2026.1740308 (PMC12979549; doi:10.3389/fnut.2026.1740308)
Supplement: Supplementary file 2 [file Table_1.docx]

**Table S1** Subgroup Analysis of TyG, TyG-BMI, and MASLD

**Table S2** Subgroup Analysis of TyG, TyG-BMI, and Liver Fibrosis

**Table S1** Subgroup Analysis of TyG, TyG-BMI, and MASLD

|  | **Variables** | **N** | **OR(95%CI)** | **p-value** | **P for interaction** |  | **OR(95%CI)** | **p-value** | **P for interaction** |
| --- | --- | --- | --- | --- | --- | --- | --- | --- | --- |
| **TyG-BMI** |  |  |  |  |  | **TyG** |  |  |  |
|  | **Gender** |  |  |  | 0.221 |  |  |  | 0.006 |
|  | Female | 4896 | 1.075(1.070,1.080) | < 0.001 |  |  | 3.972(3.220,4.900) | < 0.001 |  |
|  | Male | 2139 | 1.079(1.071,1.088) | < 0.001 |  |  | 5.357(2.867,10.011) | < 0.001 |  |
|  | **Age** |  |  |  | 0.004 |  |  |  | < 0.001 |
|  | <50 | 4456 | 1.081(1.075,1.087) | < 0.001 |  |  | 4.558(3.593,5.783) | < 0.001 |  |
|  | ≥50 | 2579 | 1.067(1.061,1.074) | < 0.001 |  |  | 3.534(2.376,5.258) | < 0.001 |  |
|  | **T2DM** |  |  |  | < 0.001 |  |  |  | < 0.001 |
|  | NO | 6185 | 1.079(1.074,1.084) | < 0.001 |  |  | 4.111(3.337,5.066) | < 0.001 |  |
|  | YES | 850 | 1.058(1.048,1.069) | < 0.001 |  |  | 3.462(2.195,5.461) | < 0.001 |  |

Note：Models are adjusted for coronary heart disease, hypertension, ALT, AST, TBIL, DBIL, IBIL, ALP, CR, UA, UREA, TG, HDL-C, and FBG.

Abbreviations：ALT, alanine transaminase；AST, aspartate transaminase；TBIL；total bilirubin；DBIL, direct bilirubin；IBIL, indirect bilirubin；ALP, alkaline phosphatase；Cr, creatinine；UA, uric acid；UREA, urea nitrogen；TG, triglycerides；HDL-C, high-density lipoprotein cholesterol；FBG, fasting blood glucose

**Table S2** Subgroup Analysis of TyG, TyG-BMI, and Liver Fibrosis

|  | **Variables** | **N** | **OR(95%CI)** | **p-value** | **P for interaction** |  | **OR(95%CI)** | **p-value** | **P for interaction** |
| --- | --- | --- | --- | --- | --- | --- | --- | --- | --- |
| **TyG-BMI** |  |  |  |  |  | **TyG** |  |  |  |
|  | **Gender** |  |  |  | 0.038 |  |  |  | 0.034 |
|  | Female | 4896 | 1.025(1.022,1.029) | < 0.001 |  |  | 1.200(0.897,1.606) | 0.219 |  |
|  | Male | 2139 | 1.035(1.026,1.044) | < 0.001 |  |  | 3.205(0.918,11.185) | 0.068 |  |
|  | **Age** |  |  |  | 0.031 |  |  |  | 0.041 |
|  | <50 | 4456 | 1.029(1.025,1.033) | < 0.001 |  |  | 1.168(0.815,1.675) | 0.398 |  |
|  | ≥50 | 2579 | 1.027(1.021,1.032) | < 0.001 |  |  | 1.453(0.791,2.670) | 0.229 |  |
|  | **T2DM** |  |  |  | 0.806 |  |  |  | 0.075 |
|  | NO | 6185 | 1.028(1.024,1.032) | < 0.001 |  |  | 1.226(0.826,1.821) | 0.313 |  |
|  | YES | 850 | 1.026(1.018,1.033) | < 0.001 |  |  | 1.305(0.745,2.286) | 0.351 |  |

Note：Models are adjusted for coronary heart disease, hypertension, ALT, AST, TBIL, DBIL, IBIL, ALP, CR, UA, UREA, TG, HDL-C, and FBG.

Abbreviations：ALT, alanine transaminase；AST, aspartate transaminase；TBIL；total bilirubin；DBIL, direct bilirubin；IBIL, indirect bilirubin；ALP, alkaline phosphatase；Cr, creatinine；UA, uric acid；UREA, urea nitrogen；TG, triglycerides；HDL-C, high-density lipoprotein cholesterol；FBG, fasting blood glucose
